# Enhanced Nitrification in Yellow Clay Improves In Situ Water Purification for Eel Aquaculture: A Preliminary Assessment

**DOI:** 10.3390/microorganisms14051126

**Published:** 2026-05-15

**Authors:** Lin Yuan, Liting Cheng, Guangnian Yuan, Chao Liu, Zhiwen Song

**Affiliations:** School of Environmental and Municipal Engineering, Qingdao University of Technology, Qingdao 266520, China; yuanl1225@126.com (L.Y.); chengliting03@163.com (L.C.); yxy_yb@163.com (G.Y.); liu.chao@qut.edu.cn (C.L.)

**Keywords:** *Anguilla japonica*, natural clay carrier, bioaugmentation, in situ water quality purification, microbial community structure, nitrifying microorganisms

## Abstract

To address the issues of high water exchange rates and significant negative environmental impacts associated with eel aquaculture, this study explored the use of yellow clay as a carrier for nitrifying bacterial communities. By pre-enhancing the nitrification capacity of the yellow clay, we aimed to improve the control of inorganic nitrogen in the aquaculture water. Three experimental groups were established: NF-YCA (nitrifying-functional yellow clay-added eel aquaculture system); NN-YCA (non-nitrifying yellow clay-added eel aquaculture system); and YC-F (yellow clay-free eel aquaculture system, blank control). The NF-YCA group had zero water exchange, while the YC-F and NN-YCA groups underwent water exchange equivalent to 28.36 times the system volume. Nitrification was most pronounced in the NF-YCA group, where both mean and peak concentrations of total ammonia nitrogen and nitrite nitrogen were lower than in the YC-F and NN-YCA groups, whereas nitrate nitrogen concentrations in the NF-YCA group were significantly higher than in the other two groups. No significant differences were observed in the survival rate and specific growth rate of elvers among the three systems during the experiment. High-throughput sequencing results revealed that Pseudomonadota and Bacteroidota were the most dominant phyla across all systems. However, the bacterial community structure in NF-YCA was more abundant and stable, and nitrification-related genera, such as *Nitrosomonas*, were detected in high abundance in this system. The preliminary results demonstrate that the eel aquaculture system with enhanced yellow clay nitrification function, can effectively maintain water quality without water exchange, highlighting its potential for practical application.

## 1. Introduction

The *Anguilla* is the sole genus within the family Anguillidae of the order Anguilliformes. It is known to comprise 19 species that spawn in the open sea [1]. Due to its high nutritional value and unique reproductive pattern, eels of the genus *Anguilla* are among the characteristic species cultivated in freshwater aquaculture worldwide. China introduced artificial eel farming from Japan in the 1970s. After more than 50 years of development, it has become the world’s largest eel producer, with aquaculture output ranking among the top globally. The main aquaculture zones of China are primarily Guangdong Province, Fujian Province, and Hainan Province [2]. According to data from the Food and Agriculture Organization (FAO) Global Fishery and Aquaculture Statistics, China’s eel aquaculture production reached approximately 292,000 tons in 2023. The genus *Anguilla* comprises several commercially valuable species, including *A. japonica*, *A. anguilla*, *A. rostrata*, *A. australis*, and *A. reinhardtii*, with *A. japonica* being the most commercially valuable cultured eel species in East Asia [3]. In addition, China, Canada, and several other countries have chosen *A. rostrata* for commercial aquaculture [4].

In eel aquaculture, the accumulation of total ammonia nitrogen (TAN) and nitrite nitrogen (NO_2_^−^-N), resulting from the decomposition of excreta, residual feed, and feces, exerts significant toxic effects on the growth of cultured organisms [5]. TAN is primarily present as either unionized ammonia (NH_3_) or the ionized ammonium ion (NH_4_^+^). NH_3_ is known to be the more toxic form, as it can readily diffuse across cell membranes and exert toxic effects on fish. Upon entering the fish’s body, NH_3_ directly impairs hemoglobin’s oxygen-carrying capacity and disrupts enzymatic metabolic pathways [6]. This dual effect leads to metabolic dysfunction, immunosuppression, and ultimately triggers a cascade of toxic reactions that culminates in central nervous system damage [7]. Nitrite is formed when ammonia is oxidized by ammonia-oxidizing bacteria (AOB) and ammonia-oxidizing archaea (AOA) under aerobic conditions, a process known as nitritation [8]. Nitrite entering aquatic animals convert hemoglobin into methemoglobin, causing suffocation and potentially leading to death [9]. Nitrite-oxidizing bacteria (NOB) convert nitrite into nitrate [10].

The main aquaculture modes currently used for *Anguilla* include pond polyculture, cage culture, and high-density intensive pond culture. These modes typically employ water exchange methods to reduce the concentration of inorganic nitrogen (ammonia and nitrite) in the aquaculture water. The daily water exchange volume accounts for approximately 10% to 20% of the total aquaculture water body (daily drainage can reach several thousand tons), which not only wastes water resources but can also have negative environmental impacts [11]. To mitigate these challenges and ensure the sustainability of eel aquaculture, effective waste management strategies are essential. Consequently, various water treatment methods have been developed and continuously refined in recent decades to address these issues. In recent years, high-density eel farms have increasingly adopted recirculating aquaculture systems (RASs) as efficient solutions for ex situ water treatment [12]. RAS was first applied to eel farming as early as 1990 [13]. Although RAS offers significant advantages in water conservation and nutrient recycling, its widespread adoption in the aquaculture industry remains limited due to substantial technical and economic constraints [14]. Furthermore, RASs present limitations for eel larvae due to their relatively low capacity to handle the organic load associated with a slurry-type diets [15].

In contrast to RAS, in situ water treatment methods, such as biofloc technology (BFT) offer a simpler approach by directly removing pollutants within aquaculture tanks, making them suitable for eel aquaculture. In situ purification has garnered widespread attention due to its higher efficiency and minimal environmental disturbance and is widely applied in the remediation of soil and water bodies [16,17,18]. The removal of ammonia and nitrites in biofloc systems is dependent mainly on the activities of heterotrophic, chemolithoautotrophic bacteria (e.g., nitrifiers) and microalgae [6]. Given this biological complexity, maintaining optimal biofloc concentrations through careful monitoring and management is crucial for system performance and risk reduction. Besides biofloc, in situ biofilm aquaculture systems (in situ BFSs) constitute a management strategy employed in the growth of aquatic organisms to improve efficiency in aquaculture tanks. The common method for removing ammonia and nitrite from aquaculture water in in situ BFSs is through the nitrification process carried out by nitrifying microorganisms [19,20]. Nitrifying microorganisms are chemoenergetic autotrophic microorganisms that rely primarily on the oxidation of inorganic nitrogen compounds to obtain the energy required for survival. Compared to heterotrophic bacteria that obtain energy by oxidizing organic matter, these characteristics lead to slow proliferation rates and relatively long generation cycles [9]. Since nitrifying microorganisms exhibit attached growth characteristics, within an in situ BFS, the substrate becomes a crucial factor for microbial growth, proliferation, and biofilm formation. However, the cost of substrates can substantially increase aquaculture production expenses.

In Chinese eel ponds, the application of yellow clay is a common practice. This approach helps create an environment that more closely resembles the eels’ natural habitat, reducing stress response, stabilizing water quality, and promoting the growth of beneficial microorganisms [21,22]. These effects are conducive to eel health and contribute to enhanced farming efficiency. This study evaluates the feasibility of constructing an in situ water purification system by enhancing the nitrification performance of yellow clay for eel aquaculture, utilizing methods such as water quality analysis and high-throughput sequencing. The specific objectives of this study were to (1) evaluate the feasibility of using yellow clay as a carrier for nitrifying bacterial communities and determine whether pre-enhancement of its nitrification performance can improve ammonia and nitrite removal in eel aquaculture systems; (2) compare the nitrification performance of pre-enhanced versus natural yellow clay; and (3) investigate the successional dynamics of bacterial communities associated with both pre-enhanced and natural yellow clay over the course of eel aquaculture and elucidate their correlations with water quality parameters.

## 2. Materials and Methods

### 2.1. Methods for Enhancing the Nitrification Function of Yellow Clay

The method for enhancing the nitrification function of yellow clay was described by Wang et al. [23]. The principle underlying this method involves modulating the supply of energy substrates (Ammonium chloride and sodium nitrite) to control the relative abundances of ammonia-oxidizing microorganisms and nitrite-oxidizing bacteria during the enrichment of nitrifying microorganisms, thereby preventing the occurrence of elevated peak concentrations of total ammonia nitrogen (TAN) and nitrite nitrogen (NO_2_^−^-N) in eel aquaculture. The cultivation procedure was as follows: A total of 25 kg yellow clay was placed in the tank containing 50 L water supplemented with 0.5% (*v*/*v*) nitrifying microbial inoculants (Qingdao Haiyisheng Environmental Technology Co., Ltd., Qingdao, China), trace element solution (0.1%, *v*/*v*) and yeast extract (0.01%, *w*/*v*). A stirrer (170–180 rpm) was used to fully suspend the yellow clay in the water. A total of 250 g of sodium nitrite (NaNO_2_) was added in five equal portions of 50 g each. Each subsequent portion was added when the nitrite nitrogen concentration dropped below 0.5 mg/L. Finally, 75 g of Ammonium chloride (NH_4_Cl) was added after the concentration of TAN fell below 0.5 mg/L, marking the completion of the cultivation process. Throughout the cultivation period, temperature and dissolved oxygen (DO) were maintained at 28 ± 1.0 °C and 4.5–6.0 mg/L, respectively.

### 2.2. Design of Experimental Eel Aquaculture Tank

**Figure 1:**
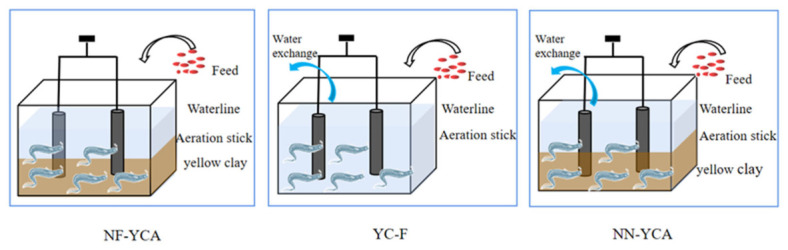
Schematic diagram of the elver cultivation system. Note: Aeration rods were used to maintain dissolved oxygen (DO) levels. NF-YCA received no water exchange throughout the experiment, while YC-F and NN-YCA were subjected to periodic water changes (see text for details).

The experiment was conducted from March to May 2025 in Qingdao, China, with an aquaculture duration of 63 days. A total of nine plastic experimental tanks (45 cm × 25 cm × 35 cm each) were used in this study. All tanks were sterilized with a 5% potassium permanganate solution prior to use. Each tank was equipped with two nano-aeration pipes (12 cm long) placed at the bottom to maintain dissolved oxygen levels (Figure 1).

In this experiment, three treatments were established with three replicates each: (1) NF-YCA (nitrifying-functional yellow clay-added eel aquaculture system); (2) NN-YCA (non-nitrifying yellow clay-added eel aquaculture system); (3) YC-F (yellow clay-free eel aquaculture system, blank control). Each aquaculture tank was provided with aeration at a rate of 22 L/min. The initial physical parameters were as follows: temperature 19.1 ± 0.5 °C, pH 8.2 ± 0.07, dissolved oxygen 9.13 ± 0.23 mg/L.

### 2.3. Management of the Aquaculture Process

Elvers of the species *A. japonica* were obtained from a hatchery in Guangdong Province, China. The Average length and weight of the elver were 9.5 ± 0.5 cm and 2.0 ± 0.1 g, respectively. Twelve elvers were stocked in each tank. Mortalities were removed and replaced timely during the experiment to maintain a stable stocking density of 600 individuals/m^3^, which is a conventional density for industrial eel aquaculture. The experimental diet was obtained from a commercial feed company in Zhongshan City, Guangdong Province. Elvers were fed once daily, with the daily ration set at approximately 6% of their total body weight. Throughout the 63-day experimental period, NF-YCA received no water exchange but was periodically replenished to compensate for water loss due to evaporation and sampling. YC-F and NN-YCA underwent a daily 20% water change starting on day 6. As water quality continued to deteriorate, the water exchange rate was increased to 50% on day 12 and maintained at this level until the experiment concluded.

### 2.4. Analytical Methods

#### 2.4.1. Analysis of Water Physicochemical Parameters

Water temperature, DO, and pH were measured using a portable multiparameter water quality analyzer (HQ30D, Hach Company, Loveland, CO, USA). The concentrations of TAN and nitrite nitrogen (NO_2_^−^-N) were determined via Nano reagent spectrophotometry (HJ 535-2009 [24]) and N-(1-naphthyl) ethylenediamine spectrophotometry (GB 7493-87 [25]), respectively. Nitrate nitrogen (NO_3_^−^-N) was analyzed by UV spectrophotometry (HJ/T 346-2007 [26]), and total phosphorus (TP) was measured using the molybdenum blue method (GB 11893-89 [27]). Throughout the experiment, TAN, NO_3_^−^-N and NO_2_^−^-N were monitored daily alongside temperature, pH, and DO, whereas TP were quantified every seven days. All measurements were conducted in accordance with national standards and repeated three times to ensure the reproducibility of the results.

#### 2.4.2. Calculation of Survival Rate and Specific Growth Rate

At the end of the experiment, body weight (g) and survival number of elvers were recorded, based on which the following parameters were calculated:

(1) $$\mathrm{Survival}\;\mathrm{Rate}\;(\%):\;\;SR\;=\;(N_{f}/N_{0})\;\times \;100\%,$$

(2) $$\mathrm{Specific}\;\mathrm{Growth}\;\mathrm{Rate}\;(\%):\;SGR=\frac{\ln W_{t}-\ln W_{0}}{t}\times 100\%.$$

where *N*_0_ and *N_f_* are the initial and final numbers of eels, respectively; *W*_0_ and *W_t_* are the initial and final mean body weights (g), respectively; and *t* is the duration of the rearing period (days).

#### 2.4.3. DNA Extraction and High-Throughput Sequencing Method

Samples for DNA extraction included: the initial yellow clay, the nitrification-enhanced clay, and the clay or suspended solids collected from the nine eel aquaculture tanks on days 35 and 63 of the experiment. DNA was extracted using an EZNA^®^ Soil DNA Kit (OMEGA, Miami, FL, USA) following the manufacturer’s instructions. DNA concentration and purity were determined using a P360 ultra-micro spectrophotometer (IMPLEN, Munich, Germany). The bacterial 16S rRNA gene V3–V4 region was amplified using primers 338F (5′-ACTCCTACGGGGAGGCAGCAG-3′) and reverse primer 806R (5′-GGACTACHVGGGGTWTCTAAT-3′) [28]. Sequencing and analysis were performed at Shanghai Meiji Biopharmaceutical Technology Co., Ltd., Shanghai, China. Bioinformatic analysis of OTUs at a 97% similarity was performed using USEARCH v11. Alpha diversity analysis of sample sequences was performed using the Qiime 1.91 platform against the silva138.2/16S_bacteria database, with a confidence level of 0.7 for species classification. Samples were rarefied to the minimum sequence depth. Raw sequencing data are available in the NCBI Sequence Read Archive (SRA) under accession No. PRJNA1398766.

### 2.5. Statistical Analysis

Data are presented as mean ± SD. The mean was calculated for the 63-day measurement results of each sample, and this mean was used as the representative value for intergroup comparisons. Prior to analysis, the Shapiro–Wilk test was used to verify the normality of the data, and the Levene test was used to verify homogeneity of variances. For indicators that met the criteria for normal distribution (Shapiro–Wilk *p*-value > 0.05) and homogeneity of variances (Levene *p*-value > 0.05), one-way ANOVA was used to compare differences among the three groups, and Tukey’s HSD test was used for post hoc comparisons. For indicators that did not meet the assumptions of normality (Shapiro–Wilk *p*-value < 0.05), the Kruskal–Wallis H nonparametric test was used to compare differences among the three groups. A *p*-value of ≤0.05 was considered statistically significant. All statistical analyses were performed using SPSS 27.0 software. Raw experimental data are provided as Supplementary Material for archival purposes only (File S1).

## 3. Results

### 3.1. Water Quality

**Figure 2:**
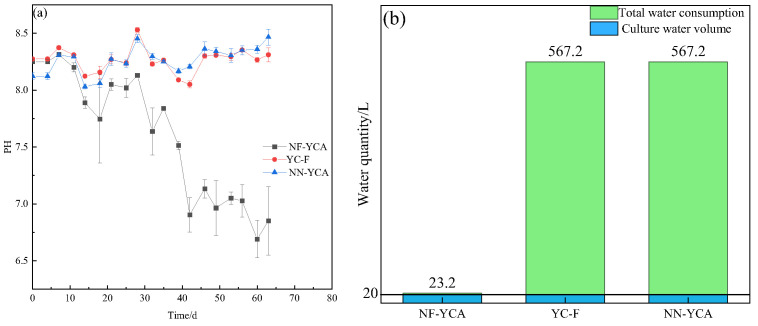
pH variations and water consumption in different eel aquaculture systems. Data are expressed as means (n = 3). Error bars represent standard errors. (**a**) pH variations, (**b**) water consumption.

Water quality parameters for the three experimental groups over the entire culture period were shown in Table 1. Unlike the NF-YCA group, which received no water exchange, the YC-F and NN-YCA groups underwent extensive water changes to maintain ammonia and nitrite nitrogen at levels suitable for eel growth. The total water consumption is equivalent to 28.36 times the volume of the aquaculture system. Details of water exchange and replenishment for three groups are presented in Figure 2. DO concentrations varied among the three culture systems, and significant differences were observed (*p*-value < 0.05). pH remained stable in all systems during the initial phase of the experiment. After 35 days, the pH of NF-YCA showed a downward trend. By the end of the experiment, the pH of NF-YCA was significantly lower than those of NN-YCA and YC-F (*p*-value < 0.05). The average concentrations of TAN and NO_2_^−^-N in the NF-YCA group were significantly lower than those in the YC-F and NN-YCA groups, while the average concentration of NO_3_^−^-N was significantly higher (*p*-value < 0.05).

### 3.2. Changes in Inorganic Nitrogen Concentrations During Eel Aquaculture

**Figure 3:**
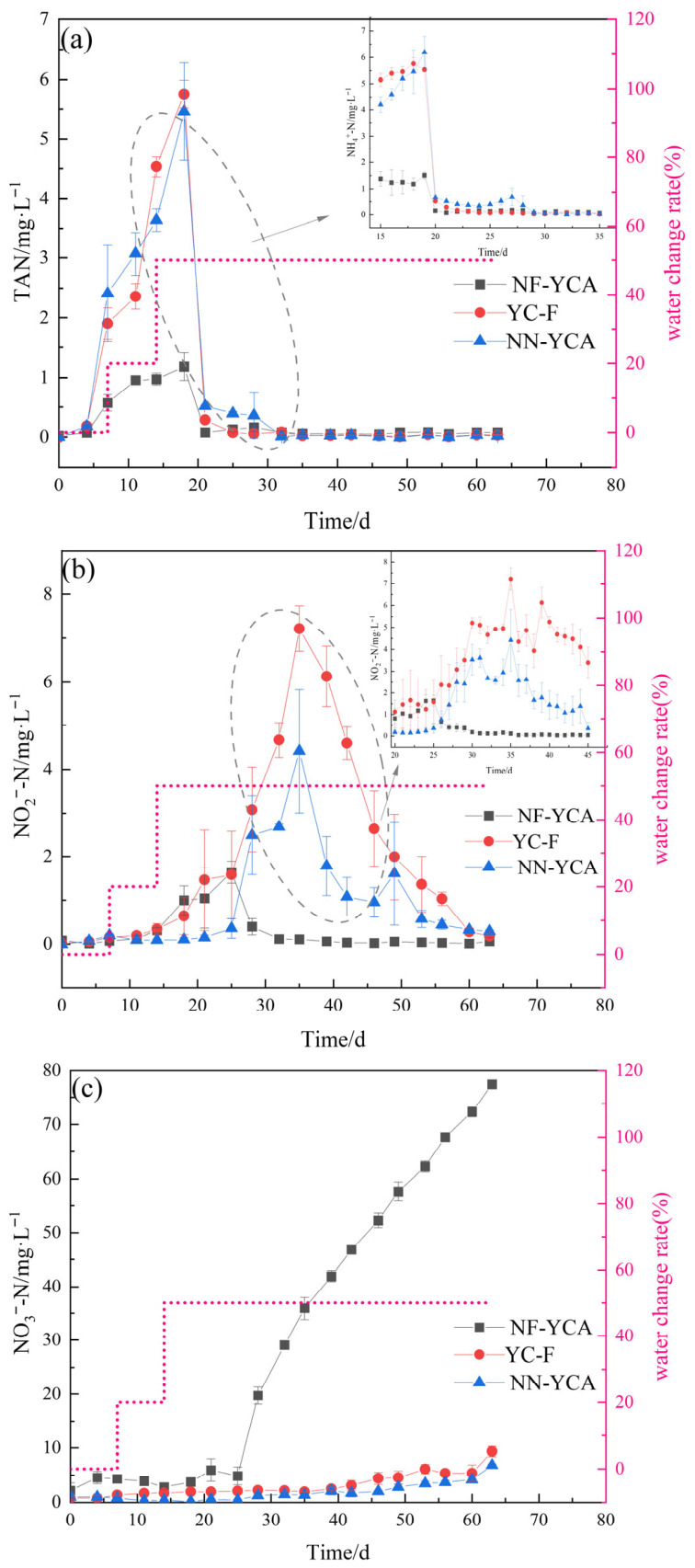
Concentrations of inorganic nitrogen in different eel aquaculture systems over the aquaculture period. Data are presented as means ± SD (n = 3). Concentrations of (**a**) TAN, (**b**) NO_2_^−^-N and (**c**) NO_3_^−^-N (mg·L^−1^).

The three groups exhibited distinct patterns of inorganic nitrogen dynamics throughout the experiment. Initially, the TAN concentration increased in all three systems (Figure 3a). On day 7, the TAN concentration in NF-YCA was significantly lower than those NN-YCA and YC-F (*p*-value < 0.05). Peak TAN concentrations in all three systems occurred simultaneously on day 19, with the peak value in NF-YCA being significantly lower than the other two groups (*p*-value < 0.05). Thereafter, TAN concentrations in NN-YCA and YC-F began to decline, driven by extensive water exchange and the gradual development of nitrification activity in the yellow clay. By day 28, TAN concentrations in NF-YCA no longer differed significantly from those in NN-YCA and YC-F (*p*-value > 0.05). Compared to the TAN concentration in the system, the NO_2_^−^-N concentration showed a delayed response in all three systems (Figure 3b). In NF-YCA, the NO_2_^−^-N concentration peaked on day 25, then declined and stabilized at a low level (<0.05 mg/L). In NN-YCA and YC-F, NO_2_^−^-N concentrations reached their peaks on day 35 and subsequently declined. Specifically, the NO_2_^−^-N concentration in NF-YCA remained significantly lower than those in NN-YCA and YC-F at the end of the experiment (*p*-value < 0.05). The NO_3_^−^-N concentrations in NF-YCA remained stable during the first three weeks (Figure 3c), then increased rapidly to 77.47 mg/L by the end of the experiment, whereas those in NN-YCA and YC-F remained consistently low throughout (<10 mg/L).

### 3.3. Growth Performance of Eel

By the end of the experiment, the average body length and weight of eels across all groups were 16.94 ± 0.59 cm and 4.07 ± 0.09 g, respectively (Table 2). Among the three groups, eels in the NF-YCA system exhibited the best growth parameters, reaching a final average weight of 4.1 ± 0.5 g, although this value was not statistically different from the other groups (*p*-value > 0.05). No significant differences in survival rate were observed among the groups, with the YC-F group showing the numerically highest survival (75.2 ± 4.7%). Specific growth rates were low across all systems, with no significant differences were observed (*p*-value > 0.05).

### 3.4. Bacterial Community Structure in Yellow Clay and Suspended Solids from Eel Aquaculture Systems

**Figure 4:**
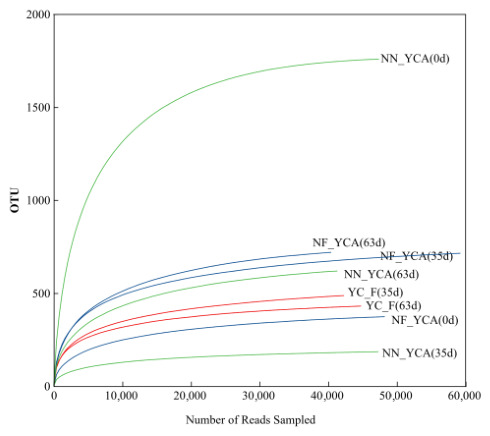
Rarefaction curves of bacterial communities in yellow clay and suspended solids under different eel aquaculture. Note: Sequencing data for the YC-F group are available only for Day 35 and Day 63.

Analysis of the rarefaction curves (Figure 4) shows that, as sequencing depth (number of reads) increases, the number of operational taxonomic units (OTU) in all samples rises rapidly before leveling off. In particular, although the NN-YCA (0 d) sample exhibited the highest species richness, its curve reached a plateau at a relatively low sequencing depth. After quality control filtering, the number of valid sequences ranged from 54,576 to 72,697, with a total of 472,836 valid sequences obtained for subsequent bioinformatics analysis. Bacterial alpha diversity, including OTU numbers and diversity indices, in yellow clay and suspended solids are presented in Table 3. All samples had Good’s coverage values of 0.9970, indicating sufficient sequencing depth. This experiment identified a total of 2830 valid OTUs. Significant differences in OTU numbers were observed among the three groups. At the beginning of the experiment, the OTU numbers in the NN-YCA (1745) were significantly higher than those in the NF-YCA (364), approximately 4.8 times higher. However, OTU numbers in the NN-YCA declined sharply during the experiment, reaching 182 by the end a reduction of 89.6%. By the end of the experiment, OTU numbers in the NF-YCA and NN-YCA had converged to similar levels. In contrast, OTU numbers in the YC-F remained relatively stable throughout the experimental period. At the beginning of the experiment, the Shannon, Simpson, and Chao indices for the NN-YCA were markedly higher than those for the NF-YCA. Specifically, the NN-YCA exhibited a Shannon index 2.02 times higher than the NF-YCA, a Simpson index of only 0.01 (indicating high evenness/diversity), and a Chao index 4.25 times higher than that of the NF-YCA. By day 35, the NF-YCA showed increased diversity, with its Shannon and Chao indices rising by 49.0% and 82.6% to 4.56 and 760.94, respectively, and its Simpson index decreasing to 0.03. Conversely, the NN-YCA exhibited a marked loss of diversity: its Shannon index fell by 67.1% to 2.03, its Chao index dropped by 87.9% to 213.71, and its Simpson index increased sharply from 0.01 to 0.29. The YC-F displayed intermediate values, both lower than those of the NF-YCA. By the experiment’s end, the NF-YCA maintained high Shannon (4.48) and Chao (763.88) indices, stable from day 35. Conversely, the NN-YCA’s Chao index recovered to a moderate level. The Simpson indices for all groups remained consistently low.

**Figure 5:**
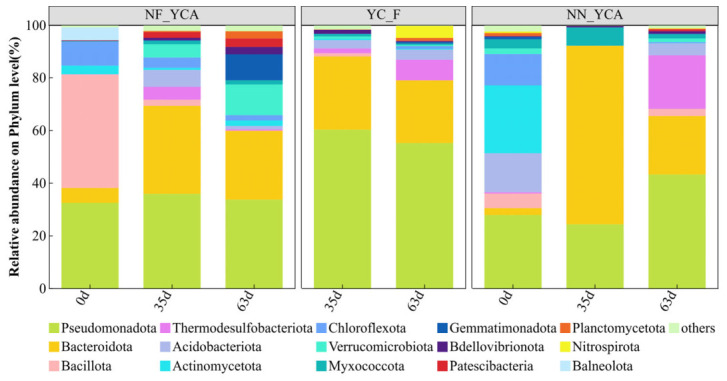
Phylum-level bacterial community composition in yellow clay and suspended solids under different eel aquaculture. Note: Sequencing data for the YC-F group are available only for Day 35 and Day 63.

**Figure 6:**
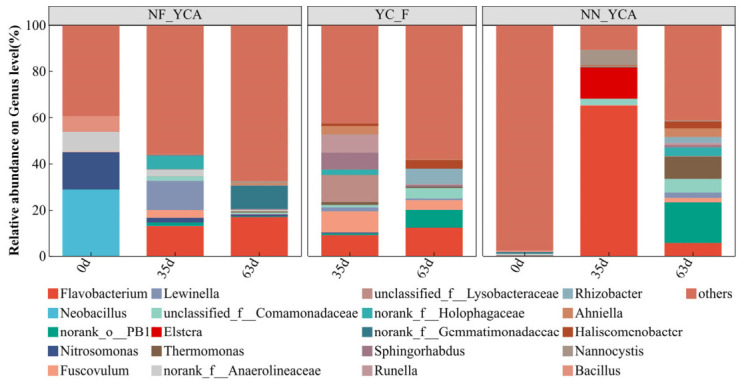
Genus-level bacterial community composition in yellow clay and suspended solids under different eel aquaculture. Note: Sequencing data for the YC-F group are available only for Day 35 and Day 63.

A total of 42 phyla and 869 genera of bacteria were detected across all samples in this experiment. At the phylum level, the three experimental groups exhibited relatively similar community structures (Figure 5), with Pseudomonadota, Bacteroidota, Bacillota, Thermodesulfobacteriota, and Acidobacteriota as the top five most abundant phyla. Among these, Pseudomonadota was the most dominant phylum across all samples, while Bacillota was primarily detected in the initial samples of NF-YCA. As the experiment progressed, the relative abundance of Bacteroidota and Thermodesulfobacteriota increased markedly. At the genus level, *Flavobacterium* exhibited a marked increase in relative abundance as the experiment progressed (Figure 6), eventually becoming the dominant genus within the aquaculture systems. *Neobacillus* was detected almost exclusively in the initial samples of the NF-YCA group, but was scarcely detected thereafter. Notably, the relative abundance of *Norank_o_PB19* was significantly lower in NF-YCA than in YC-F and NN-YCA.

The distribution results of bacterial genera associated with nitrogen cycling during the aquaculture period are shown in Table 4. Two genera of AOB, *Nitrosomonas* and *Nitrosospira*, and two genera of NOB, *Nitrospira* and *Nitrococcus*, were identified. *Nitrosomonas* was detected in high abundance in the initial samples of the NF-YCA, and it began to appear in the YC-F and NN-YCA as the experiment progressed (Figure 6). For instance, in NF-YCA, the relative abundance of *Nitrosomonas* declined from 16.287% at the beginning to 2.088% at mid-culture, and further to 0.856% by the end of the experiment. Meanwhile, *Nitrospira* was detected at a relative abundance of 0.485% in the NN-YCA samples. During aquaculture, its relative abundance declined to undetectable levels but recovered to half of the initial value by the end of the experiment. In contrast, a continuous enrichment of this genus was observed in the NF-YCA group. The highest relative abundance of *Nitrospira* (4.556%) was recorded in the YC-F group at the final sampling point.

**Figure 7:**
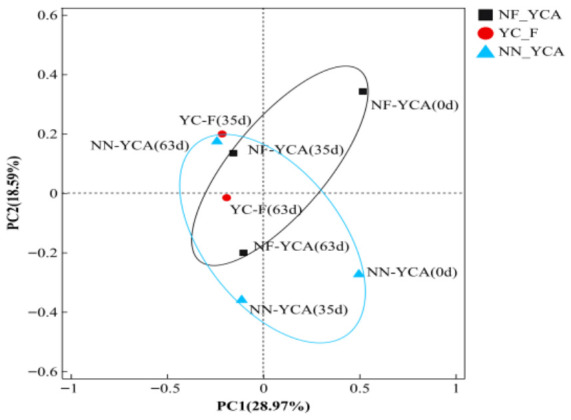
PCoA of bacterial communities at the genus level in different eel aquaculture systems. Note: Sequencing data for the YC-F group are available only for Day 35 and Day 63.

Principal coordinate analysis (PCoA) was conducted at the genus level based on Bray–Curtis distance to assess the beta diversity of bacterial communities in yellow clay and suspended solids (Figure 7). PC1 and PC2 explained 28.97% and 18.59% of the total variation, respectively accounting for 47.56% cumulatively. Most samples clustered tightly in the left region of the plot. In contrast, at the start of the experiment, samples from both the NF-YCA and NN-YCA groups showed a clear shift toward the right-hand side of the plot, forming a distinct cluster separate from the other samples. The PCoA ordination results indicate that the aquaculture system shaped the bacterial community structure.

## 4. Discussion

Throughout the entire eel farming process, all water quality parameters were suitable for elver cultivation [29,30]. The DO levels in this experiment were higher than those in other aquaculture settings [31,32]. No hypoxic events were observed during the experiment. In addition, previous studies [33] have pointed out that supersaturated oxygen can reduce the toxicity of ammonia to fish. This may explain why no large-scale eel mortality occurred during the early stage of ammonia accumulation in this experiment. The gradual decrease in alkalinity of the NF-YCA system during elver cultivation is directly related to the nitrification process. Because nitrification produces acidic end products [34]. The YC-F and NN-YCA groups maintained a pH of approximately 8.3 throughout the cultivation period, which was attributed to the frequent water changes. SHI et al. [35] indicates that at pH values below 9.0, the ammonia removal capacity of nitrifying bacteria shows no significant impact. This aligns with the observation in this study that after ammonia oxidation function was established, the ammonia-nitrogen concentrations in all three systems remained at low levels. Nitrification is significantly influenced by temperature [36,37,38]. Previous studies have indicated [39,40], nitrifying bacteria achieve their maximum nitrification reaction rate around 30 °C. In this experiment, the temperature was lower than in other nitrification processes, resulting in a prolonged period required for the complete establishment of ammonia oxidation functionality. The lower temperature may explain the accumulation of ammonia nitrogen observed during the early stages of cultivation.

Zero-water-change elver rearing reduces the harm caused by water changes to fish larvae. However, it also leads to a gradual increase in TAN and nitrite levels in the water, as well as the accumulation of nitrate. Results indicate that TAN, NO_2_^−^-N, and NO_3_^−^-N concentrations in all treatments remained within the acceptable ranges for elver growth. However, the water quality of the NF-YCA is significantly superior to that of the YC-A and NN-YCA. Meanwhile, multiple water changes were performed in both the YC-A and NN-YCA systems, with the total water consumption amounting to approximately 28.36 times the volume of water used for aquaculture. In the NF-YCA, water replacement is unnecessary, significantly reducing water consumption and lowering the risk of disease outbreaks [14]. The addition of feed and the decomposition of excrement lead to the accumulation of harmful substances such as ammonia and nitrites within the aquaculture system [41]. The nitrifying-functional yellow clay of NF-YCA plays a significant role in stabilizing water quality. In the YC-F and NN-YCA groups, the initial populations possessing ammonium oxidation capabilities were relatively low, limiting their nitrification capacity. Consequently, these groups relied primarily on frequent water exchange to control TAN concentrations, whereas the NF-YCA system maintained low ammonia levels without water exchange due to its enhanced nitrification activity. This led to peak TAN concentrations in the YC-F and NN-YCA systems (Figure 3a). Nitrifying bacteria are known to be environmentally sensitive and slow-growing. However, as the experiment progressed, ammonia oxidation capacity was gradually established in all three systems. Coupled with the regular removal of uneaten feed and feces, this led to a significant improvement in water quality by the end of the experiment [42]. After three weeks of aquaculture, the TAN concentration remained low, with no significant differences among treatments (*p*-value > 0.05). Accumulation of NO_2_^−^-N was also observed across the three systems. This nitrite accumulation occurs because ammonia-oxidizing microorganisms (AOM) grow faster than NOB, preventing the timely conversion of nitrite to nitrate [19]. In the NF-YCA system, NOB were enriched more rapidly (Table 4), which facilitated nitrite conversion and led to lower nitrite peaks compared to YC-F and NN-YCA (Figure 3b). Nitrates, which primarily originate from the nitrification of ammonia and nitrite, can accumulate in aquaculture systems [43]. In the NF-YCA, the absence of water exchange led to significant nitrate accumulation, resulting in nitrate concentrations that were significantly higher than those in YC-F and NN-YCA (Figure 3c). However, nitrate is generally considered to have low toxicity and little effect on the health of aquatic organisms.

Since the eels selected for the experiment were sourced from the same broodstock, their growth potential is expected to be identical [44]. Over the nine-week experimental period, no significant differences were observed in the rearing conditions among the various treatments. This suggests that normal growth and development of elvers can be achieved through large-scale water changes in the aquaculture system, even without the addition of yellow clay with nitrification capabilities. Compared to previous studies, the survival rate of eels in this experiment was higher than that observed in natural pond farming [45,46] and lake farming [47]. Although most eels in natural rivers have the habit of burrowing into fine sediments [48,49], it appears that adding yellow clay to the aquaculture system does not affect the growth of elvers. After the addition of yellow clay, although the water quality in the NN-YCA system was slightly superior to that in the YC-F system, no significant differences were observed in the survival rate or specific growth rate of elvers between the two systems.

Quantifying the extent to which bacteria adhere to clay particles is a challenging task [50]. In the experiment, the removal efficiency of ammonia nitrogen and nitrite nitrogen in each experimental group was used as an indirect measure of microbial activity and attachment efficiency. Clay minerals provide nutrients and habitats for microorganisms, in turn, microorganisms can induce the formation of clay minerals [51]. The physical properties of minerals (such as morphology and roughness) and their chemical properties (such as surface charge) are the primary intrinsic factors influencing bacterial colonization [52]. Furthermore, the properties of the clay not only influence initial adhesion but also regulate the subsequent metabolic activity of the bacteria [53]. Interactions between microorganisms and clay minerals promote the nitrogen cycle [51].

The removal of toxic nitrogenous compounds in aquaculture systems is a microbially mediated process, closely linked to the diversity, abundance, and functional roles of the bacterial community [54]. A sample coverage of 0.99 demonstrates that the sequencing depth is sufficient [23]. Microbial diversity serves as a measure of microorganisms’ adaptive capacity under changing environmental conditions [23,55]. In the present study, following the addition of nitrifying yellow clay, the NF-YCA system exhibited higher Shannon and Chao1 indices, along with a lower Simpson index. This indicates that the bacterial community in the NF-YCA aquaculture water was more complex and stable [56]. Consequently, the NF-YCA system demonstrated greater resilience to water quality fluctuations, highlighting its capacity to maintain stable conditions during environmental disturbances.

Pseudomonadota are widely recognized as the most abundant phylum in nitrogen-cycling microbial communities [57,58] and play a crucial role in promoting water quality [59]. They are also frequently detected in mature biofloc technology systems [22]. Consistent with these characteristics, Pseudomonadota exhibited the highest relative abundance across all samples in the present study, indicating that active nitrogen cycling, mediated by the microbial community, was occurring within the water body. Bacteroidota are recognized as a potential probiotic phylum in aquaculture systems [60,61]. In accordance with this, a relatively high proportion of Bacteroidota was observed in the microbial communities of farmed eel water in the present study, which is consistent with previous findings by Yang [62], who reported that supplementing elver diets with Bacillus sp. positively influenced survival rates. Many genera within the phylum Bacillota (e.g., Lactobacillus and Bacillus) are widely used as probiotics in aquaculture [63]. In the present study, Bacillota exhibited a relative abundance of 43.08% in the initial NF-YCA sample, but only 5.44% in the initial NN-YCA sample. Throughout the subsequent cultivation period, the relative abundance across all samples remained low, ranging from 0.02% to 2.85%. This range is comparable to the abundance of Bacillota reported by Argentino et al. in natural water bodies [64]. Previous studies have demonstrated a significant positive correlation between the abundance of Thermodesulfobacteriota and TAN removal efficiency [65]. Consistently, in this work, the relative abundance of Thermodesulfobacteriota was higher in environments with lower TAN concentrations. In contrast to this autotrophic nitrification-related phylum, Acidobacteriota and Planctomycetes both reported as predominant components of the eel gut microbiota [62] were rarely detected in initial water samples but appeared only after the cultivation period. Nitrospirae is a phylum that contains the genus *Nitrospira*, which encompasses many known NOB [66]. At the conclusion of the experiment, the YC-F group was found to contain Nitrospirae, possibly due to an enrichment effect triggered by earlier high concentrations of nitrite. *Flavobacterium* is a well-known fish pathogen commonly isolated from freshwater aquaculture environments and is responsible for substantial economic losses in the industry [67].

*Nitrosomonas* is a common AOB genus in wastewater treatment systems [68]. In the present study, its relative abundance was higher in the NF-YCA than in the other. This likely facilitated the prompt oxidation of TAN, resulting in a lower peak ammonia concentration compared to the other groups. As TAN levels subsequently decreased, the relative abundance of *Nitrosomonas* in NF-YCA declined. However, the continuous accumulation of nitrate within the NF-YCA indicated that the microbial community retained high nitrification activity throughout the experiment [54]. *Nitrospira* was the NOB most frequently detected in this study. It is worth noting that at the end of the experiment, *Nitrospira* accounted for 4.56% of the relative abundance in YC-F, which was significantly higher than in the NF-YCA and NN-YCA. This difference was not caused by fluctuations in sequencing depth but rather truly reflected the ecological differentiation of the nitrifying microbial communities under different aquatic environments. On the one hand, as a typical oligotrophic nitrifying bacterium [69], *Nitrospira* prefers clean water environments with low levels of suspended solids, high dissolved oxygen, and minimal organic interference. On the other hand, the intermediate concentration of nitrite in the YC-F group was higher, providing *Nitrospira* with a more abundant supply of nitrite substrate, which further enhanced its growth and reproductive advantage [70]. In addition, 16S rRNA gene sequencing (DNA-based) cannot distinguish between live cells (including dormant cells and metabolically active cells, whether growing or not) and dead cells, which may lead to false-positive results [71]. It is worth noting that the results of this study, which are based on high-throughput sequencing, reflect only the relative composition of NOB within the overall microbial community and do not directly correspond to its absolute biomass. There are inherent differences among the samples in terms of sample volume, substrate properties, and total microbial biomass. Specifically, the YC-F group did not contain yellow mud; the suspended matter was formed gradually through the decomposition of uneaten feed and feces during eel farming. The concentration of suspended matter was far lower than that of yellow mud. Although high-throughput sequencing results indicated that the YC-F group had a higher relative abundance of NOB at 63 days, the low concentration of suspended matter did not significantly affect the nitrite concentration in the aquaculture system. Therefore, the nitrite content in the YC-F group was higher than that in the NF-YCA and NN-YCA groups. Similarly, FU K [56] reported that *Nitrospira* could dominate aerobic activated sludge in both batch and continuous flow reactors. Despite being detected as a core microbial genus in other aquaculture systems [72,73], the role of *Lewinella* in water quality improvement remains unclear. PCoA revealed that the first two samples (NF-YCA (0 d) and NN-YCA (0 d)) were clearly separated from all other samples. Differences in bacterial communities among the three aquaculture systems gradually decreased over time, likely driven by the stabilization of environmental conditions and the gradual establishment of the nitrifying bacterial community [34].

In summary, the addition of nitrifying yellow mud to aquaculture systems has positive implications for water conservation and water quality improvement. In situ addition of nitrifying yellow clay effectively removes ammonia nitrogen and nitrite from elver aquaculture water, optimizes the microbial community structure, increases microbial community richness and stability, and facilitates water remediation. This study focused solely on the microbial community structure of the aquaculture water, but the addition of nitrifying microorganisms could be ingested by elver, potentially altering their intestinal microbial community structure. Therefore, future research should further investigate the gut microbiome of aquatic organisms, based on analysis of changes in the microbial structure of the aquaculture environment. Under the conditions of this experiment, the survival rate of eels was relatively low. The low survival rate can be attributed to two main factors. Firstly, the limited space in the experimental aquaculture system restricted the eels’ mobility; secondly, the small sample size meant that the death of a single individual could significantly affect the calculated survival rate, introducing a degree of systematic error. Future research will involve larger-scale experiments with improved experimental designs to further elucidate the factors affecting eel survival. High-throughput sequencing can only reflect relative community abundances. It neither provides absolute quantification nor effectively distinguishes between active, dormant, and dead cells. Consequently, this limits our ability to assess the functional status of nitrifying bacteria. Future studies will incorporate metatranscriptomics to characterize the metabolic activity of nitrifying bacteria at the gene expression level, thereby providing a more comprehensive and accurate understanding of their ecological functions.

## 5. Conclusions

1. Sequencing results demonstrated that yellow clay can serve as a carrier for nitrifying bacterial communities. The pre-enhanced yellow clay exhibited superior nitrification performance and stronger inorganic nitrogen purification capacity, achieving zero water exchange, whereas the YC-F and NN-YCA groups consumed a total of 28.36 times the system volume in water.
2. Compared with pre-enhanced yellow clay, natural yellow clay showed inferior effectiveness in becoming a carrier for nitrifying bacterial communities. However, the NN-YCA group displayed lower ammonia and nitrite concentrations than the YC-F group, indicating that the addition of yellow clay can gradually improve nitrification performance as eel aquaculture proceeds, thereby enhancing the inorganic nitrogen control capacity of the aquaculture system.
3. As eel aquaculture progressed, the bacterial community compositions of pre-enhanced nitrifying yellow clay and natural yellow clay became more similar. The nitrifying bacteria in the natural yellow clay group were continuously enriched, facilitating nitrification reactions and reducing ammonia and nitrite nitrogen concentrations.

## 6. Patents

Some contents of this research have been patented (Chinese Patent: ZL 201910999131.8; PCT Patent: PCT/CN2020/079551).

## Figures and Tables

**Table 1:** Temperature, pH, DO, and nitrogen concentrations in different Eel Aquaculture systems over 63 days.

	NF-YCA	YC-F	NN-YCA
Temperature (°C)	21.0 ± 2.2 ^a^ (17.9–24.5)	21.1 ± 2.3 ^a^ (18.0–24.9)	21.0 ± 2.5 ^a^ (17.7–24.9)
DO (mg/L)	8.07 ± 0.63 ^a^ (7.11–9.27)	8.50 ± 0.41 ^b^ (7.73–9.34)	8.41 ± 0.42 ^c^ (7.8–9.34)
pH	7.60 ± 0.57 ^a^ (6.5–8.32)	8.26 ± 0.11 ^b^ (8.02–8.39)	8.26 ± 0.12 ^b^ (8.01–8.54)
TAN (mg/L)	0.27 ± 0.40 ^a^ (0.01–1.58)	0.86 ± 1.66 ^b^ (0–6.03)	0.92 ± 1.62 ^b^ (0–6.58)
NO_2_^−^-N (mg/L)	0.26 ± 0.42 ^a^ (0.01–1.93)	1.96 ± 1.98 ^b^ (0.02–7.6)	0.90 ± 1.11 ^c^ (0–6.05)
NO_3_^−^-N (mg/L)	23.38 ± 17.57 ^a^ (0.55–77.70)	3.09 ± 2.01 ^b^ (0.55–10.03)	1.85 ± 1.70 ^b^ (0.07–7.08)
TP (mg/L)	0.57 ± 0.43 ^a^ (0–1.30)	0.11 ± 0.09 ^ab^ (0–0.26)	0.04 ± 0.04 ^b^ (0–0.11)

Note: Data are presented as mean ± SD. Different superscript letters indicate significant differences among treatments within the same row.

**Table 2:** Growth performance of eels after 63 days in different eel aquaculture systems.

	NF-YCA	YC-F	NN-YCA
Total number of eel (ind)	49	48	54
Survival number of eel (ind)	36	36	36
Final length (cm)	17 ± 0.6	16 ± 2	16 ± 2
Initial weight (g)	2.0 ± 0.1	2.0 ± 0.1	2.0 ± 0.1
Final weight (g)	4.1 ± 0.5	3.96 ± 0.2	3.8 ± 0.3
Survival rate (%)	74.3 ± 10.1	75.2 ± 4.7	67.6 ± 9.3
Specific Growth Rate (%)	1.16 ± 0.1	1.12 ± 0.1	1.11 ± 0.1

Note: No significant differences in growth performance were observed in the different eel aquaculture systems.

**Table 3:** Bacterial community diversity indices in yellow clay and suspended solids under different eel aquaculture.

		NF-YCA			YC-F			NN-YCA	
	0 d	35 d	63 d	0 d	35 d	63 d	0 d	35 d	63 d
Reads	60,593	72,697	57,491	Nm	55,333	54,576	56,672	57,602	57,872
OTUs	364	667	719	Nm	484	425	1745	182	618
Shannon	3.06	4.56	4.48	Nm	4.06	4.23	6.17	2.03	4.20
Simpson	0.12	0.03	0.04	Nm	0.04	0.03	0.01	0.29	0.05
Chao	416.78	760.94	763.88	Nm	547.57	465.78	1772.25	213.71	659.33
Good’s coverage	0.9981	0.9971	0.9974	Nm	0.9977	0.9984	0.9970	0.9990	0.9976

Note: Nm, sequencing not conducted.

**Table 4:** Relative abundance of nitrifying bacteria in different eel aquaculture systems.

	NF-YCA			YC-F			NN-YCA		
	0 d	35 d	63 d	0 d	35 d	63 d	0 d	35 d	63 d
*Nitrosomonas* (%)	16.29	2.09	0.856	Nm	0.47	0.04	0.02	0.02	0.05
*Nitrosospira* (%)	0.060	0.02	0	Nm	0	0	0	0	0
*Nitrospira* (%)	0	0.03	0.09	Nm	0.01	4.56	0.49	0	0.24
*Nitrococcus* (%)	0.008	0.01	0	Nm	0	0	0	0	0

Note: Nm, sequencing not conducted.

## Data Availability

The original contributions presented in this study are included in the article/Supplementary Material. Microbial sequencing data have been uploaded to public databases; the accession numbers are listed in the text. Further inquiries can be directed to the corresponding author.

## Supplementary Materials

The following supporting information can be downloaded at: https://www.mdpi.com/article/10.3390/microorganisms14051126/s1, File S1: Raw data (Origin file) containing all original experimental data (not presented or referenced in the main text).

## Abbreviations

The following abbreviations are used in this manuscript:

AOA	Ammonia-oxidizing Archaea
AOB	Ammonia-oxidizing Bacteria
AOM	Ammonia-oxidizing Microorganisms
BFSs BFT	Biofilm Aquaculture Systems Biofloc Technology
DO	Dissolved Oxygen
NOB	Nitrite-oxidizing Bacteria
OTUs	Operational Taxonomic Units
PCoA	Principal Coordinate Analysis
RASs	Recirculating Aquaculture Systems
TP	Total Phosphorus
